# The DNA damage repair-related lncRNAs signature predicts the prognosis and immunotherapy response in gastric cancer

**DOI:** 10.3389/fimmu.2023.1117255

**Published:** 2023-06-29

**Authors:** Zidan Zhao, Tsz Kin Mak, Yuntao Shi, Huaping Huang, Mingyu Huo, Changhua Zhang

**Affiliations:** ^1^Digestive Diseases Center, The Seventh Affiliated Hospital of Sun Yat-sen University, Shenzhen, China; ^2^Guangdong Provincial Key Laboratory of Digestive Cancer Research, The Seventh Affiliated Hospital of Sun Yat-sen University, Shenzhen, Guangdong, China

**Keywords:** DNA damage repair, gastric cancer, lncRNA signature, immune infiltration, immunotherapy

## Abstract

**Background:**

Gastric cancer (GC) is one of the most prevalent cancers, and it has unsatisfactory overall treatment outcomes. DNA damage repair (DDR) is a complicated process for signal transduction that causes cancer. lncRNAs can influence the formation and incidence of cancers by influencing DDR-related mRNAs/miRNAs. A DDR-related lncRNA prognostic model is urgently needed to improve treatment strategies.

**Methods:**

The data of GC samples were obtained from The Cancer Genome Atlas (TCGA) and Gene Expression Omnibus (GEO) datasets. A total of 588 mRNAs involved in DDR were selected from MSigDB, 62 differentially expressed mRNAs from TCGA-STAD were obtained, and 137 lncRNAs were correlated with these mRNAs. Univariate Cox regression and least absolute shrinkage and selection operator (LASSO) regression analyses were used to develop a DDR-related lncRNA prognostic model. Based on the risk model, the differentially expressed gene signature A/B in the low-risk and high-risk groups of TCGA-STAD was identified for further validation.

**Results:**

The prognosis model of 5 genes (AC145285.6, MAGI2-AS3, AL590705.3, AC007405.3, and LINC00106) was constructed and classified into two risk groups. We found that GC patients with a low-risk score had a better OS than those with a high-risk score. We found that the high-risk group tended to have higher TME scores. We also found that patients in the high-risk group had a higher proportion of resting CD4 T cells, monocytes, M2 macrophages, resting dendritic cells, and resting mast cells, whereas the low-risk subgroup had a greater abundance of activated CD4 T cells, follicular helper T cells, M0 macrophages, and M1 macrophages. We observed significant differences in the T-cell exclusion score, T-cell dysfunction, MSI, and TMB between the two risk groups. In addition, we found that patients treated with immunotherapy in the low-RS score group had a longer survival and a better prognosis than those in the high-RS score group.

**Conclusion:**

The prognostic model has a significant role in the TME, clinicopathological characteristics, prognosis, MSI, and drug sensitivity. We also discovered that patients treated with immunotherapy in the low-RS score group had a better prognosis. This work provides a foundation for improving the prognosis and response to immunotherapy among patients with GC.

## Introduction

Gastric cancer (GC) is one of the most prevalent cancers worldwide; it ranks fourth in incidence (5.6%) and fifth in morbidity (7%) among all cancers (1). Early GC can be resected endoscopically, and advanced GC is treated with standard D2 radical surgery, adjuvant/neoadjuvant chemotherapy, targeted therapy, and immunotherapy; however, the overall treatment outcome of GC remains unsatisfactory due to the difficulty in detecting early GC and the high incidence of distant metastases in advanced GC (2). Therefore, more effective diagnostic, prognostic, and therapeutic sensitivity biomarkers are desired.

DNA damage repair (DDR) is a complicated process for signal transduction that plays a significant part in ensuring genomic stability. DNA damage may be caused by both external and endogenous hazardous chemicals (such as heavy metals, ionizing radiation, and oxygen radicals) (3). Both nitrite and Helicobacter pylori have been identified as risk factors for GC. The formation of nitrous acid or N-nitrosamine in the stomach by nitrite and infection with Helicobacter pylori can induce DNA damage in gastric epithelial cells (4), leading to activation of proto-oncogenes or inactivation of tumor suppressor genes. To protect genomic integrity, cells have evolved a remarkable DDR system. Previous studies have identified DDR mechanisms, including direct reverse repair, base excision repair, nucleotide excision repair, mismatch repair, and homologous recombination (HR)/nonhomologous end joining (NHEJ) double-strand break (DSB) repair (5). More than 500 related proteins are engaged in DDR-related pathways. According to previous research, BRCA2, ATM, RAD51, and ATR mutations in the DRR pathway are prevalent in GC and are strongly associated with overall survival (OS) (6–8). Additionally, the DDR-related pathway is crucial for regulating the treatment response of cancer (9), suggesting that DRR-related genes have a significant role as diagnostic, prognostic, and therapeutic biomarkers for GC.

Long noncoding RNAs (lncRNAs) are key regulators at the transcriptional and posttranscriptional levels of the genome and are dysregulated in a variety of malignant tumors, making them useful as diagnostic, prognostic, and therapeutic biomarkers (10). Recent studies have demonstrated that lncRNAs can influence the formation and incidence of cancers by influencing DDR-related mRNAs/miRNAs (11). In nasopharyngeal cancer tissues, linc00312 was dramatically downregulated, and patients with high expression of linc00312 had longer OS and improved short-term radiation effectiveness (12). Mechanistically, linc00312 binds to DNA-PKcs and suppresses the phosphorylation of the AKT-DNA-PKcs axis, thereby influencing the NHEJ repair process. The lncRNA SLC26A4-AS1 suppresses the expression of several DSB repair genes, and its low expression level is significantly associated with poor prognosis in thyroid cancer patients (13). Tumour suppressors of the miR-34 family (miR-34a, miR-34b, and miR-34c) play a crucial role in the DNA damage response (14). Another study used dynamic Boolean network analysis and revealed that lncRNA GAS5 regulates miR-34c by targeting E2F1 and affects the ATM/p38 MAPK signaling pathway to inhibit GC proliferation (15). However, few lncRNAs interacting with DRR-related genes have been studied in GC.

From The Cancer Genome Atlas (TCGA) and Gene Expression Omnibus (GEO) databases, we obtained transcriptome, clinicopathological, and OS data for GC in this research. The Molecular Signatures Database (MSigDB) was collected for a total of 588 genes involved in DDR. Differential analysis and correlation analysis were used to establish the coexpression network of lncRNAs-mRNAs involved in DDR. The predictive efficacy of a GC prognosis model based on five DDR-related lncRNAs was validated using an internal cohort, an external cohort, and cluster analysis. In addition, comprehensive bioinformatics studies were carried out to investigate the differences between high- and low-risk groups in immune infiltration, chemotherapy, targeted medicines, and immunotherapy sensitivity. Finally, the expression levels of these five lncRNAs in GC cell lines and tissues were confirmed. This research provides a glimpse into improving the prognosis of GC patients and expanding the understanding of DDR-related lncRNAs.

## Method

### Data collection

Transcriptome sequencing data and corresponding clinical information were collected for stomach adenocarcinoma (STAD) samples from TCGA (https://portal.gdc.cancer.gov/), including tumor samples (n = 375) and normal samples (n = 32). GEO (https://www.ncbi.nlm.nih.gov/geo/) was used to gather tumor samples sequencing data, including those from the GSE15459 (n=192), GSE26253 (n=432), GSE26901 (n=109), GSE26899 (n=93), and GSE84433 (n=357) cohorts with detailed characteristic information and survival. The IMvigor210 cohort (immunotherapy cohort) expression data and clinical information were obtained from http://research-pub.gene.com/IMvigor210CoreBiologies/. The raw data downloaded above were normalized for subsequent analysis using the “Deseq2” package (16).

In addition, DDR-related genes were downloaded by searching for “DNA damage repair” in MSigDB (http://www.gsea-msigdb.org/msigdb/MSigDB/index.jsp).

### Identification of DDR-related lncRNAs and the mRNA−lncRNA coexpression network

From the “GOBP DNA REPAIR” project in the MSigDB database, a total of 588 DDR-related mRNAs were obtained **(** **Supplemental Materials Table 1****)**. The TCGA-STAD gene expression matrix was divided into an mRNA expression matrix and a lncRNA expression matrix using the Perl scripts and the “human.gtf” annotation file. Using the “limma” package in R (version 4.1.1) for differential analysis (17), 62 differentially expressed DDR-related mRNAs were identified from 588 DDR-related mRNAs (p< 0.05, |logFC| > 1.5) based on the TCGA-STAD mRNA expression matrix **(** **Supplemental Materials Table 2****)**. Based on these 62 differential DDR-related mRNAs, mRNA-lncRNA correlation analysis identified 137 DDR-related lncRNAs (P< 0.001, |Pearson correlation coefficient| > 0.5) **(** **Supplemental Materials Table 3****)**.

### Construction and validation of a DDR-related lncRNA risk model in the TCGA-STAD internal cohort

A total of 371 TCGA-STAD patients were randomly divided into a training group (n = 187) and a test group (n = 184) at a ratio of 1:1 to validate the DDR-related lncRNA signature. The R “glmnet” package was used to identify the core prognostic lncRNAs using the LASSO (least absolute shrinkage and selection operator)-Cox regression analysis, and the value of the penalty parameter (λ) was selected based on the lowest partial likelihood deviance using 10-fold cross-validation (18). When lambda (λ) was minimized, we obtained lasso models for the five prognostic lncRNAs and their corresponding model coefficients, which were multiplied by the coefficients and the corresponding lncRNA expression to calculate risk scores and conduct subsequent analyses. The calculation formula is as follows:


$$\text{Risk score}=\sum_{i=1}^{n}{\text{Coef}}_{\text{DDRrlncRNA}}\;*\;{\mathrm{Exp}}_{\text{DDRrlncRNA}}$$

The Coef_DDRrlncRNA_ is the risk coefficient of DDR-related lncRNA used to construct the formula, whereas Exp_DDRrlncRNA_ is the expression of DDR-related lncRNA in the formula. Then, we calculated each patient’s risk score. According to the median risk score, patients were categorized as high-risk (risk score more than the median) or low-risk (below the median risk score). Kaplan−Meier analysis was utilized to compare the survival rates of the high- and low-risk groups. To evaluate the sensitivity and specificity of the signature, time-dependent receiver operating characteristic (ROC) analysis was performed using the “survivalroc” package (19). Calibration curves were generated to assess whether the predicted survival rates were consistent with the actual survival rates (20). All analyses were performed in the TCGA-STAD internal training group, test group, and all samples. The independence of DDR-related lncRNA signature on OS was further explored by univariate and multivariate Cox proportional risk regression (CPHR) analyses using clinical information (age, gender, grade, stage, survival time, survival status) combined with Risk score in TCGA-STAD.

### Nomogram

Based on R (version 4.1.1), we used the calibrate functions of “rms” package (http://CRAN.R-project.org/package=rms) to construct a nomogram to predict 1-year, 3-year, and 5-year OS rates in TCGA-STAD. In the nomogram scoring system, each variable is assigned a score, and the total score for each sample is calculated by summing the scores of all variables (21). We evaluated the predictive ability of the 1, 3, and 5-year models using the Kaplan-Meier method, constructed calibration curves, and assessed the concordance between the predicted OS rate and the actual observed OS rate. The reliability of the model was evaluated using decision curve analysis (DCA) (22).

### Consensus clustering

Consensus clustering in unsupervised learning is also a widely used classification method in cancer research. The “ConsensusClusterPlus” package was used to determine the number of clusters and their stability (23). Five prognostic DDR-related lncRNAs were employed to identify and categorize molecular subgroups of patients in TCGA-STAD. Additionally, 1,000 replications were conducted to confirm categorization stability. To further explore the clinical value of consensus clustering, associations between genetic subtypes, clinicopathological characteristics, and risk models were investigated. In addition, Kaplan−Meier analysis was employed to assess the differences in survival rates between clusters. Finally, the “ggplot2” package (24) was used to visualize the differences in clusters of DDR-related lncRNAs used to construct the prognostic model.

### Gene Set Variation Analysis (GSVA)

GSVA is a nonparametric and unsupervised method that is frequently used to evaluate the biological features of different subgroups (25). GSVA is a specific type of gene set enrichment method that evaluates whether different pathways are enriched across samples by converting the expression matrix of genes across samples into an expression matrix of gene sets, essentially investigating the differences between specific gene sets across samples. GSVA evaluations are usually performed on gene sets from the MSigDB database, while customized gene sets can also be analyzed. Based on R (4.1.1), the “GSVA” and “limma” packages were utilized to search and show the differential gene sets between the high- and low-risk groups (|logFC| > 0.2 and P< 0.05).

### Validation of DDR-related risk model in external cohorts

External GC cohorts (GSE15459, GSE26253, GSE26901, GSE26899, and GSE84433) used as validation sets did not contain all DDR-related lncRNAs used for establishing risk score. It is an effective analysis method to further establish an alternate scoring system by GSVA to identify the differential gene sets of high and low-risk groups (26). Based on the risk model established by DDR-related lncRNAs, the differentially expressed gene signature A/B in the low-risk and high-risk groups of TCGA-STAD was identified to further validate the clinical application value of the risk model. Gene Signature A is a set of highly expressed genes found in the high-risk group, whereas Gene Signature B is highly expressed in the low-risk group. GSVA enrichment analysis was performed to estimate the enrichment scores of gene signatures A and B in each TCGA-STAD sample, and the RS score was calculated by subtracting the enrichment score of gene signature A from the enrichment score of gene signature B. By analyzing the discrepancies between gene signature A, gene signature B, and RS score in different risk groups, as well as the association between RS score and risk score, this method may be exploited as an alternative risk score method. Finally, RS score in the external GEO cohorts were calculated, and the Kaplan−Meier method was used to compare the OS between the high-RS score group and the low-RS score group.

### Estimation of the immune and gene mutation landscape

CIBERSORT (https://cibersort.stanford.edu/) is a common algorithm for obtaining cell composition from gene expression profiles (27). The CIBERSORT method with the LM22 gene signature was used to calculate the proportion of 22 kinds of tumor-infiltrating immune cells (TIICs) in TCGA-STAD. We compared the heterogeneity in immune cell infiltration into the tumor microenvironment between the high- and low-risk groups.

The “Maftools” R package was used to evaluate mutations in different Risk score groups (28). Cancer stem cells (CSCs) are a kind of cell identified in tumor tissues that have been associated with tumor metastasis, recurrence, and drug resistance (29). CSCs were characterized by mRNA expression-based Stemness index (mRNAsi) (30). The One-class logistic regression (OCLR), a complicated machine learning method that utilizes machine learning to extract feature sets of transgenes and epigenomes from untransformed pluripotent stem cells and their progeny, is an efficient way to assess the level of cancer differentiation (30). Using the “TCGAbiolinks” R package in R (version 4.1.1), the OCLR machine learning method was used to calculate the mRNAsi of each sample based on the level of its mRNA expression (31). Using the “limma” package, the correlation between mRNAsi and Risk score was further analyzed.

### Immunotherapy evaluation based on RS score

Tumor Mutational Burden (TMB) is the number of somatic mutations in the coding sequence (CDS) region of the longest transcript sequence per million bases (MB), which includes the total number of base substitutions and insertion/deletion mutations (32). A higher TMB may result in the generation of more neoantigens, raise the probability of T cell recognition, render immunotherapy more effective, and be associated with better outcomes with immune checkpoint inhibitors (ICIs). Microsatellite instability (MSI) refers to the instability of microsatellite (MS) sequence length due to insertion or deletion mutations during DNA replication (33). Mismatch repair (MMR) defects often cause this condition. The mutation information data of TCGA-STAD was downloaded from the TCGA database, which included sample information and the corresponding mutant genes, chromosome locations, mutant bases, mutation types, etc **(** **Supplemental Materials Table 4****)**. Based on the mutation information data, the Perl script calculates the TMB of the matching sample. In addition, we obtained MSI status assessment tables for various TCGA samples **(** **Supplemental Materials Table 5****)**. The TMB and MSI status of the two groups of samples with high and low RS scores were compared by the Wilcox test. In addition, the correlation between the RS score and TMB was calculated. Tumor Immune Dysfunction and Exclusion (TIDE, http://tide.dfci.harvard.edu/) can be applied to identify biomarkers that predict the therapeutic efficacy of ICIs (34). Higher TIDE prediction scores indicate a greater probability of immune evasion, indicating that patients are less likely to respond favorably to ICIs treatment. The T-cell-inflamed score (TIS) can predict the clinical response to immune checkpoint blockade based on existing antitumor immunity in the tumor microenvironment (TME) (35). The TIDE, MSI, T-cell dysfunction, and exclusion scores were obtained for each TCGA-STAD sample through the TIDE online database to assess the potential clinical efficacy of immunotherapy in different RS score groups **(** **Supplemental Materials Table 6****)**. The Wilcoxon test was applied to compare TIDE, MSI, and T-cell dysfunction and exclusion scores between different RS score groups. In addition, the TIS was calculated using the mean log2-scale normalized expression of the 18 signature genes **(** **Supplemental Materials Table 7****)**. Using the “timeROC” R package (https://cran.r-project.org/web/packages/timeROC/), we compared the prognostic values of the RS score, TIDE, and TIS and performed a time-dependent ROC curve analysis to obtain AUC. Furthermore, anti-PDL1 cohort (IMvigor210) samples were classified using the RS score. The “survival” and “ggplot2” packages were used to plot OS curves, different degrees of immunotherapy response, and the proportion of patients with or without response.

### Drug sensitivity analysis

To predict anticancer drug sensitivity in different risk groups, the anticancer drug dataset was downloaded from the Genomics of Drug Sensitivity in Cancer (GDSC) website (https://www.cancerrxgene.org/), and the “oncoPredict” package was applied to calculate the IC50 of different anticancer drugs in different risk groups (36). We screened the TCGA-STAD samples for drugs with a standardized mean IC50<1, as such samples, are considered effective for the treatment of gastric cancer, and we performed wilcox-test of drug sensitivity for these drugs in the high- and low-risk subgroups to determine the varying levels of response to drugs in patients of different risk groups.

### Cell culture and tissue samples

The normal human gastric epithelial cell line GES-1, as well as the human gastric cancer cell lines AGS, MGC-803, SNU-719, HGC-27, and MKN-28, are both accessible in our lab. **Supplemental Materials Table S8** provided the major genetic characteristics of the cell lines. All cell lines were cultured in RPMI-1640 medium (Gibco, Grand Island, NY, USA) supplemented with 1% penicillin/streptomycin and 10% fetal bovine serum. Cells were grown in 5% CO_2_ at 37°C. The experimental cells were in the logarithmic growth phase.

Twenty GC samples and matched adjacent samples from patients treated at the Seventh Affiliated Hospital of Sun Yat-sen University were frozen at -80°C. Before undergoing surgery, every patient submitted a consent letter. The Seventh Hospital of Sun Yat-sen University’s Ethical Review Committee authorized this research.

### RNA extraction and qRT−PCR validation

TRIzol was used to extract total RNA (Invitrogen). A reverse transcription kit (TaKaRa) was used to convert the RNA to cDNA. All operations were carried out by the manufacturer’s instructions. Fluorescence quantitative PCR equipment (CFX96 Touch, Bio-Rad) was used to detect the expression of lncRNAs, and the reaction conditions were carried out according to the operating instructions of the fluorescence quantitative PCR kit (SYBR Green Mix, Roche). Quantitative PCR was carried out in three duplicates per reaction. GAPDH was chosen as the internal reference of lncRNAs. The 2^-^ΔΔCT method was used for data analysis, ΔΔCt=experimental group (Ct_target-lncRNA_-Ct_GAPDH_)-control group (Ct_target-lncRNA_-Ct_GAPDH_). The amplification primer sequences of each gene and its internal reference are detailed in **Table 1**.

**Table 1:** Real-time quantitative PCR primer sequences used in this study.

DDR-related lncRNAs	Forward (5′-3′)	Reverse (5′-3′)
MAGI2-AS3	TCTTCAAGAGCCAGGGACAG	TGCAGCTCAAACTCTCCAGA
LINC00106	AGTGGTCACCTGAGATGGAGCAG	CGTCTGTCTTACGGCACGAAGC
AC145285.6	AGTGGGAGAGAATCAAGTCGGTGAA	GGCTTCTAAGGCGGTTTGAGACCTT
AL590705.3	AGAGATTTAATTGTGGTTCTGCCAAGGA	AGTTTTGGTTACAGGCTCCCAAGTG
AC007405.3	CCAGCCTTTTGGCTAAGATCAAGTGT	AGGAGATGACTACAGGAAGGGCTTT

### Statistical analysis

R Studio (R version 4.1.1), GraphPad Prism 6.0 (GraphPad Tools, San Diego, CA, USA), and PASS 15.0 software were applied for statistical analysis. Each chapter introduces particular software packages utilized by R studio. The Wilcox test was used to compare two groups, one-way ANOVA was used to compare multiple groups, Pearson correlation analysis was used to determine correlations, and Kaplan−Meier analysis was used for survival analysis. Any p< 0.05 was considered statistically significant.

## Results

The workflow of the prognostic model analysis is illustrated in **Figure S1**. This study included 1590 samples, including normal samples from TCGA-STAD (n=32) and GC samples from TCGA-STAD (n=375), as well as GC samples from GSE15459 (n=192), GSE26253 (n=432), GSE26901 (n=109), GSE26899 (n=93), and GSE84433 (n=357).

### Identification of DDR-related lncRNAs in GC

**Figure 1:**
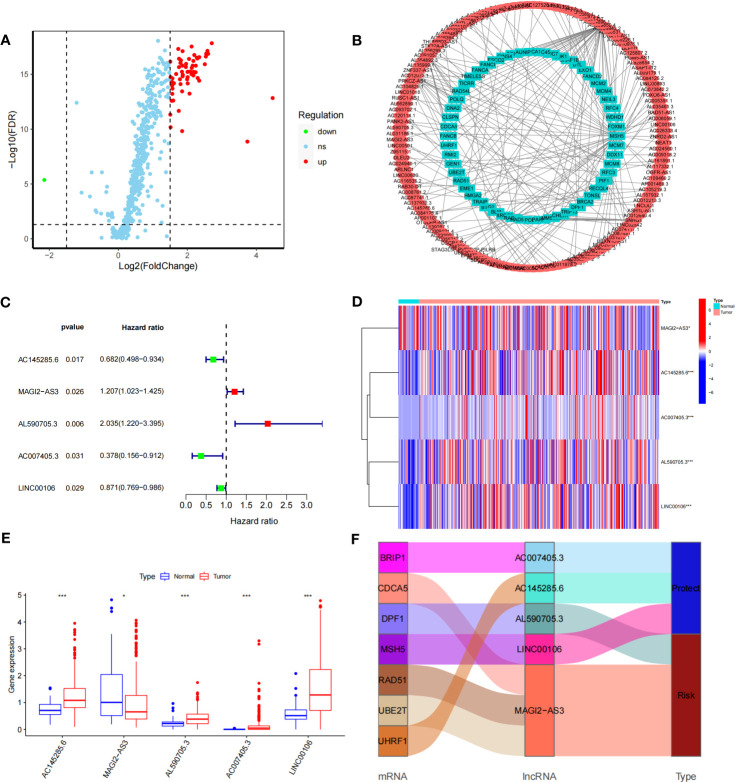
Construction of the DDR-realted mRNA's-incRNAs coexperience network and identifying prognostic DDR-realted incRNAs **(A)** Volcano plot of 558 DDR-related genes in GC. Red dots represent up-regulated genes dots represent down-regulated genes. **(B)** The co-expression network of DDR-related IncRNAs. **(C)** HR and 95% CI of the top five IncRNAs using univariate Cox regression. **(D, E)** The expression levels of five DDR-related IncRNAs in tumor and normal issues. **(F)** Sankey diagram of prognostic DDR-related IncRNAs *P<0.05, ***P<0.001.

The DDR-related gene set containing 588 genes was selected from MSigDB. After differential analysis of these genes in TCGA-STAD, 62 differentially expressed DDR-related genes were identified **(** **Figure 1A****)**, and mRNA−lncRNA coexpression network analysis was conducted **(** **Figure 1B****)**. Five DDR-related lncRNAs with prognostic values were identified using univariate analysis **(** **Figure 1C****)**. AC145285.6, AC007405.3, and LINC00106 were considered protective factors for GC prognosis, whereas MAGI2-AS3 and AL590705.3 served as risk factors. Kaplan-Meier analysis showed that patients with high expression of AC007405.3, AC145285.6, and LINC00106 had a good prognosis, while those with high expression of AL590705.3 and MAGI2.AS3 had a poor prognosis **(** **Figure S2****)**. The expression of these five chosen DDR-related lncRNAs in TCGA-STAD was further plotted using a heatmap and box line diagram **(** **Figures 1D, E**), and it was revealed that their expression was significantly different; AC145285.6, AL590705.3, AC007405.3, and LINC00106 expression was increased in GC tissues, but MAGI2-AS3 expression was decreased. The Sankey diagram described the coexpression of these prognostically significant DDR-related lncRNAs linked to their respective mRNAs **(** **Figure 1F****)**.

### Construction of DDR-related lncRNA prognostic signature

**Figure 2:**
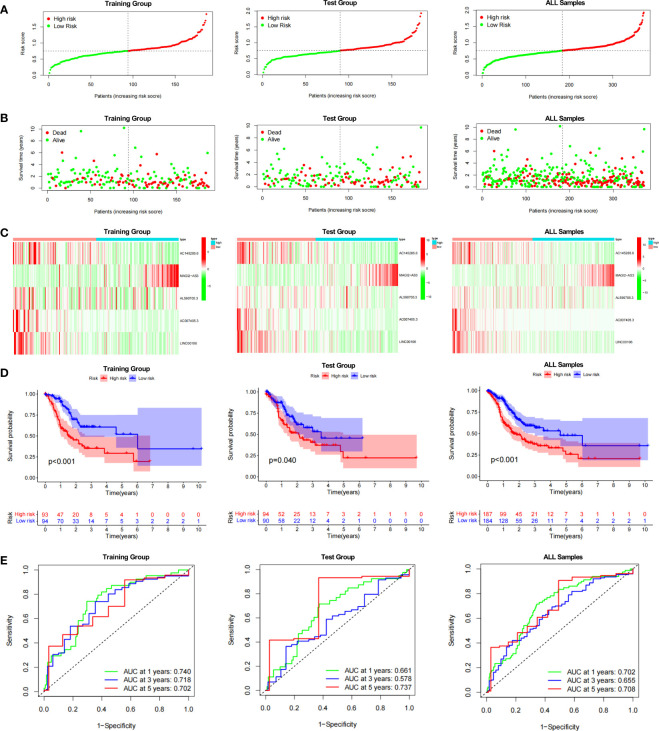
Evaluation of the DDR-related IncRNAs signature in the TCGA-STAD cohort. **(A, B)** The distribution of the risk scores and scatter plots of survival in patients in the training group, text group, and all samples. **(C)** Prognostic signature signal heatmaps in the different group **(D)** The Kaplan-Meter curve analysis of the low-and –high-risk groups in the different group. **(E)** Receiver operating characteristics (ROC) curve analysis of the signature in the diffrerent group.

The prognostic model for GC based on these five DDR-related lncRNAs was built by univariate Cox regression and LASSO regression **(** **Figure S3****)**. The formula is described in the method chapter, and the specific coefficients are shown in **Table 2**. In TCGA-STAD, GC patients were separated into a training group and a test group. Using the formula, each patient’s risk score was calculated, and patients were classified into high-risk and low-risk groups **(** **Figure 2A****)**. More deaths were observed in the high-risk group **(** **Figure 2B****)**, and a heatmap revealed the distribution of these five lncRNAs in the different risk groups **(** **Figure 2C****)**. AC145285.6, AC007405.3, and LINC00106 were higher in the low-risk group, whereas MAGI2-AS3 was elevated in the high-risk group. Additionally, survival curves were used to assess the prognostic value of the model for OS in GC. The OS rate in the high-risk group was considerably lower in the training group, test group, and overall sample **(** **Figure 2D****)**. The model’s prognostic accuracy was further assessed using ROC **(** **Figure 2E****)**. The 5-year AUCs for the training group, test group, and overall sample were 0.702, 0.737, and 0.708, respectively. Taken together, these results suggest that this DDR-related lncRNA signature is a valuable prognostic model for GC.

**Table 2:** The respective coefficients of five lncRNAs in the risk model formula.

DDR-related lncRNAs	coefficients
MAGI2-AS3	0.211108414500143
LINC00106	-0.123766252153069
AC145285.6	-0.188414949324606
AL590705.3	0.0399582944274701
AC007405.3	-0.56881526329518

### Evaluation of DDR-related lncRNA signature

**Figure 3:**
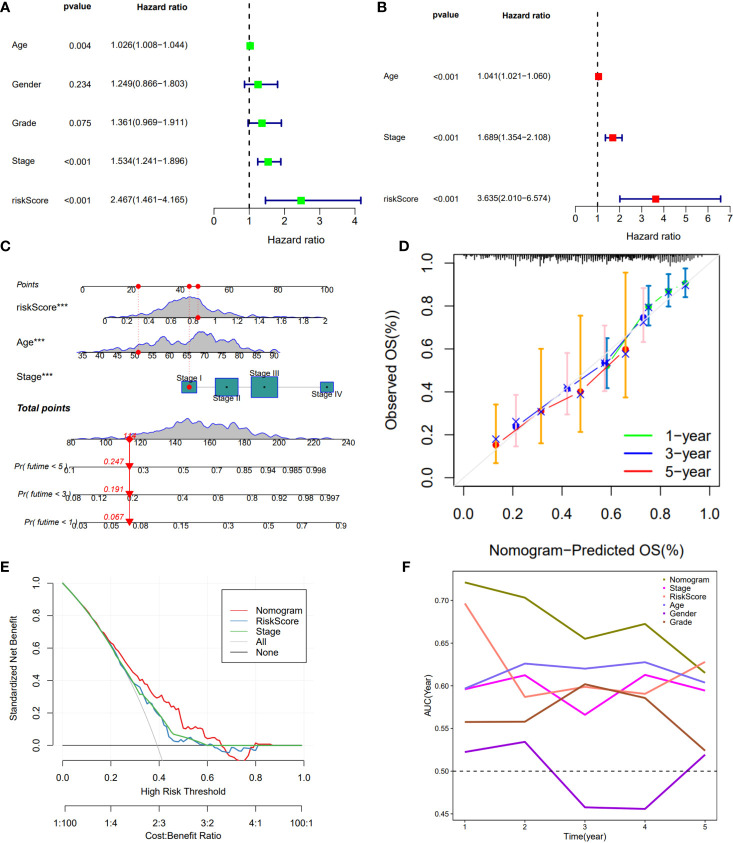
Construction of a nomogram model integrated with the risk score. **(A, B)** Univariate and multivariate Cox analyses included different clinicopathologic features. **(C)** Nomogram model for predicting the 1-, 3-, and 5 –year OS of Gc patients. **(D)** The calibration plots for 1-, 3- and 5 years in the TCGA-STAD. **(E)** Decision curve for nomogram. **(F)** Comparison of the predictive capacity of clinic pathological features and the nomogram using time- ROC analysis. ***P <0.001.

Cox regression was used to analyze whether the prognostic models constructed based on the five DDR-related lncRNAs were independent risk factors for GC. Univariate COX regression demonstrated that the risk model, tumor stage, and age could significantly affect patient prognosis (**Figure 3A**), whereas multivariate COX regression indicated that the risk model was an independent prognostic factor for GC [P<0.001, hazard ratio (HR) = 3.635, 95% confidence interval (CI) = 2.010-6.574] (**Figure 3B**). To further evaluate the efficacy of risk models for practical application, a nomogram was constructed using clinicopathological parameters and risk scores to predict 1-year, 3-year, and 5-year OS in GC patients (**Figure 3C**), with the patient’s prognosis worsening as the risk score increased. The calibration curve showed relatively good fits for OS prediction (**Figure 3D**). These results demonstrate the clinical applicability of nomograms that integrate risk models. In addition, DCA demonstrated that the nomogram was better for the risk score and stage in discriminating against patients at high risk **(** **Figure 3E****)**. The TimeROC analysis revealed that the AUC of the risk score and nomogram was higher than that of the other TCGA-STAD indicators **(** **Figure 3F****)**.

### Identification of GC subtype based on prognostic DDR-related lncRNAs

**Figure 4:**
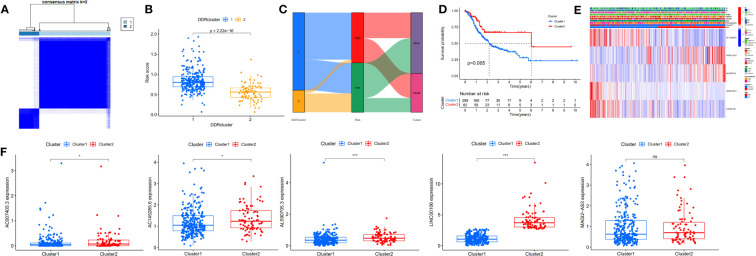
Identification of DDR-related lncRNAs subtypes in GC. **(A)** Consensus matrix heatmap defining two clusters (K=2). **(B)** Differences in risk score between two DDR clusters. **(C)** Alluvial diagram of the distribution of the subtypes in groups with different risk score and survival outcomes. **(D)** Kaplan-Meier curves for OS of the DDR-related lncRNAs subtypes. **(E)** Relationships between clinicopathologic features, low-/high-risk group, and the two lncRNAs subtypes. **(F)** Differences in the expression of five DDR-related lncRNAs among the two subtypes. NS: no significance, *P<0.05, **P<0.01, ***P<0.001.

To further assess the prognostic potential of using these five DDR-related lncRNAs in the TCGA internal cohort in different methods of prognostic model construction, we used the unsupervised clustering method to identify different regulatory patterns based on the expression levels of five DDR-related lncRNAs. K=2 divided the TCGA-STAD cohort into clusters 1 (n = 289) and 2 (n = 82) (**Figure 4A**). **Figure 4B** shows the difference in risk score between patients with two DDR clusters. **Figure 4C** shows the distribution of patients within the two DDR subtypes, two risk score groups, and survival state. The survival study revealed a substantial survival difference between cluster 1 and cluster 2, with cluster 2 exhibiting a higher survival rate (**Figure 4D**). In combination with clinicopathological data and risk models, heatmaps and box plots displaying the expression patterns of five lncRNAs in patients from distinct clusters indicated that patients in cluster 1 had a higher risk score, suggesting a worse prognosis (**Figures 4E, F**). Except for MAGI2-AS3, the remaining four DDR-related lncRNAs exhibited differential expression in two different clusters, with all of them exhibiting higher expression in cluster 2. These findings show that these five DDR-lncRNAs can more accurately represent the prognosis of GC patients.

### Validation of risk models in the external cohort

**Figure 5:**
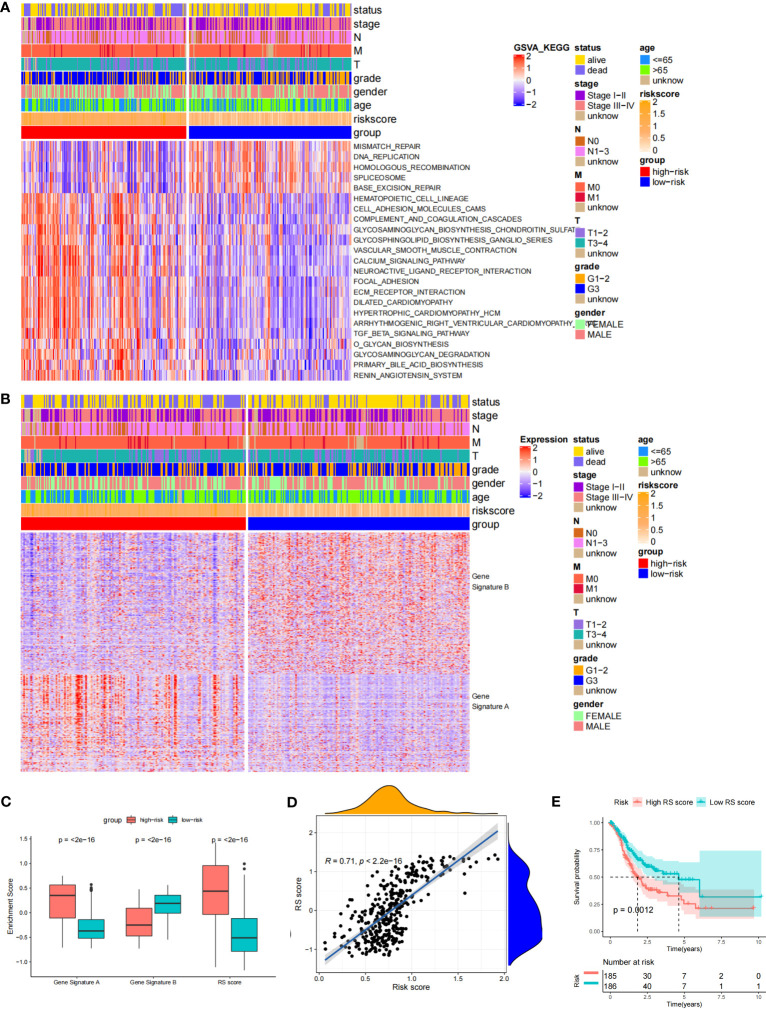
Biological characteristics in the different risk score groups and Construction of the RS score. **(A) ** Heat Map showing differentially enriched biological pathways between the low- and high-risk groups. **(B)** Heat map displaying DEG signature termed as gene signature A/B between the low- and high-risk groups. **(C)** Boxplot showing the difference in the enrichment score of gene signature A/B and the RS score between the low- and high-risk groups. **(D)** Spearman correlation analysis between RS score and risk score. **(E)** The Kaplan-Meier curve shows the OS of patients with different RS scores.

**Figure 6:**
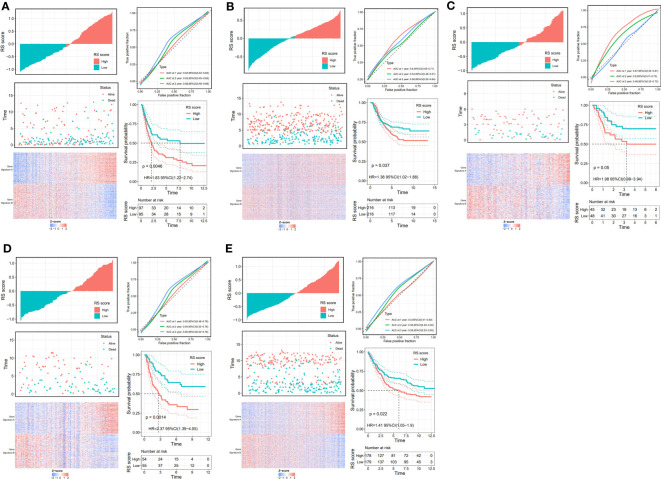
Validation of the prognostic DDR-related IncRNA signature based on RS Score. The upper left parts are distribution plots for the relationship between RS score and survival status the lower left parts are heat maps for the gene signature A/B in the cohorts; the upper right parts are ROC curve for the RS score in the different external cohorts; the lower right parts are survival curves between high-and low-RS score groups. **(A)** GSE15459; **(B)** GSE26253; **(C)** GSE26253; **(D)** GSE26901; **(E)** GSE84433.

Through the signal pathway enrichment analysis of the high-low risk group of TCGA-STAD, it was discovered that the high-risk patients had rich signal pathways associated with tumor development and metastasis, such as cell adhesion molecules, focal adhesion, extracellular matrix (ECM) receptor interaction, and TGF beta pathway. Additionally, we found that a series of DDR processes, including mismatch repair, DNA replication, homologous recombination, spliceosomes, and base excision repair, were enriched in the low-risk group. Indirectly, these results reflect the accuracy of the risk model constructed by DDR-related lncRNAs and the more aggressive molecular features of high-risk patients (**Figure 5A**). To understand the efficacy of the risk score, gene signatures A and B were applied to discern between high- and low-risk groups, with gene signature A being expressed in the high-risk group and gene signature B being expressed in the low-risk group (**Figure 5B**). The risk gene signature score (RS score) was calculated using the expression differential between gene signatures A and B in the high- and low-risk groups. Both gene signatures A and B and the RS score were significantly different between the high-risk and low-risk groups (**Figure 5C**), and there was a significant correlation between the risk score derived from DDR-related lncRNAs and the RS score (**Figure 5D**). Kaplan−Meier analysis confirmed that high RS scores were associated with poor prognosis (**Figure 5E**). The above results suggest that the RS score method is an alternative risk score model. To further confirm the predictive efficacy of the alternative model, the prognostic value of the RS score was validated in GSE15459, GSE26253, GSE26901, GSE26899, and GSE84433. In each of the five external cohorts, we calculated each patient’s RS score using the same method. According to the calculated median value of the RS score, patients were divided into the high-RS score and low-RS score groups. The results of the five external cohort survival analyses uniformly indicated that patients with a high RS score had a poor prognosis. Further ROC curve analysis revealed that the alternative model could reliably predict patient prognosis (**Figure 6**).

### Landscape immune infiltration and gene mutation in different risk score groups

**Figure 7:**
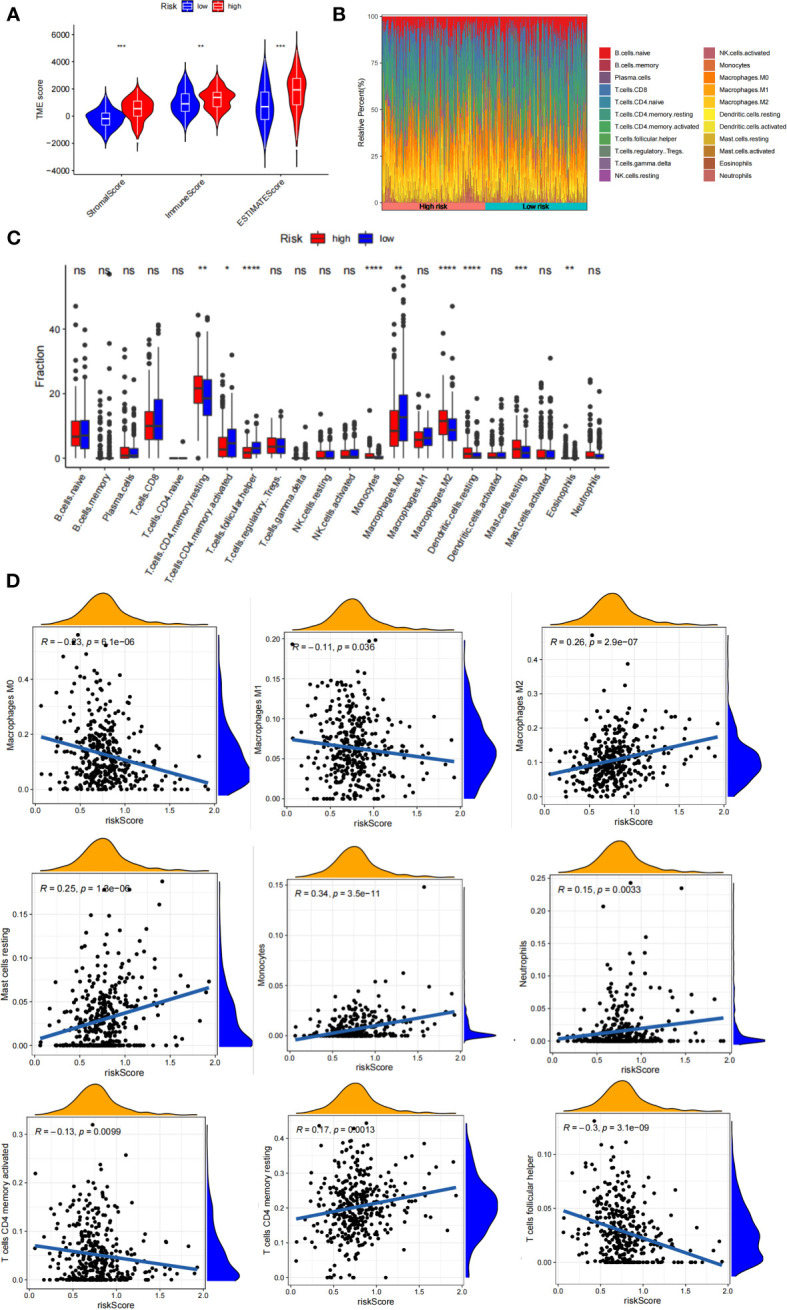
Immune infiltration in two risk score subgroups. **(A)** Differences in tumor microenvironment between high-and low-risk groups. **(B)** Composition of immune cells in different subgroups. **(C)** The relative immune infiltration score of 22 immune cells between low- and high-risk groups. **(D)** relationships between risk score and different immune cells. NS: no significance, *P<0.05, **P<0.01, ***P<0.001, ****P<0.0001.

The tumor microenvironment plays a vital role in the development of GC. Based on the estimate algorithm, we analyzed immune scores, stromal scores, and ESTIMATE scores in TCAG-STAD and found that the high-risk group tended to have higher scores (**Figure 7A**). Further analysis was conducted to examine the immune microenvironment status of the low- and high-risk groups by CIBERSORT, and bar graphs were used to visualize the infiltration levels of 22 immune cells (**Figures 7B, C**). The findings revealed that the patients with high-risk scores had a higher proportion of resting CD4 T cells, monocytes, M2 macrophages, resting dendritic cells, and resting mast cells, whereas the low-risk patients had a greater abundance of activated CD4 T cells, follicular helper T cells, M0 macrophages, and M1 macrophages. Additionally, correlation analysis of the risk score and immune cell infiltration revealed that M2 macrophages, resting mast cells, monocytes, neutrophils, and memory CD4 T cells increased as the risk score increased, whereas M0 and M1 macrophages, activated CD4 memory T cells, and follicular helper T cells decreased (**Figure 7D**). We evaluated gene mutations to develop a deeper insight into the immunological characteristics of different risk subgroups. We found the 20 genes with the highest mutation rates in the high-risk and low-risk subgroups (**Figure S4A-B**). These genes had a greater mutation rate in the low-risk group. The results revealed that missense mutations were the most frequent type of mutation. The mutation rates of TTN, TP 53, and MUC 16 were the most common and were above 25% in both groups. In addition, we observed a negative correlation between the risk score and the CSC index (**Figure S4C**). Given that risk scores are statistically correlated with the majority of immune cell infiltration levels, gene mutations, and CSC index, the immunotherapy outcomes of patients may be affected.

### Evaluation of the immunotherapy response based on RS score

**Figure 8:**
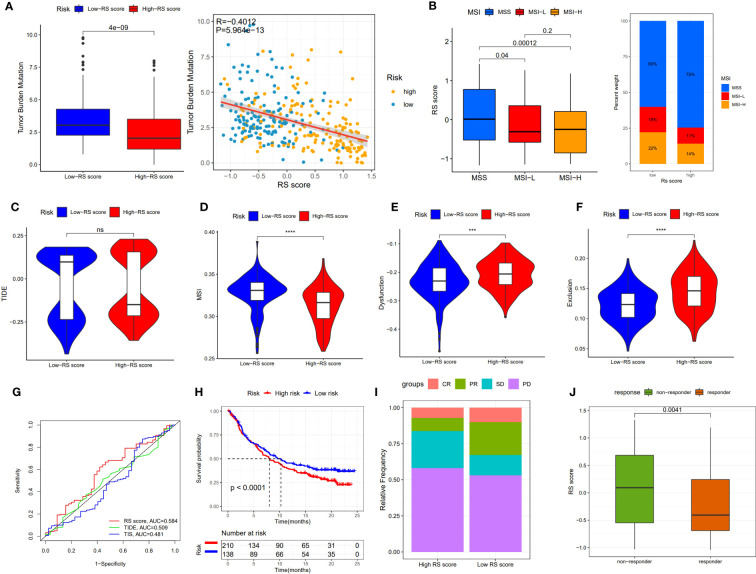
The prognostic value of RS score in immunotherapy from TCGA-STAD and IMvigor210 cohort. **(A)** TMB score in different RS score subgroups and the correlation between TMB, high-/low-risk groups, and RS score. **(B)** Relationship between RS score and MSI. **(C-F)** TIDE, T cell exclusion, T cell dysfunction, and MSI in different RS score subgroups, respectively. **(G)** ROC analysis of RS score, TIDE, and TIS on OS in TCGA-STAD. **(H)** Kaplan-Meier curve and log-rank test compare the OS of patients with low or high RS score in the IMvigor210 cohort. **(I)** Bar plot displaying the relative frequency of different clinical response subgroups in the low or high RS score group. **(J)** Boxplot demonstrating the RS score difference between the response group and the non-response group. NS: no significance, ***P<0.001, ****P<0.0001.

Although immunotherapy has significant efficacy and few serious adverse events, only some malignant tumor patients are sensitive to immunotherapy, and molecular subtypes are still required to pinpoint populations that respond to immunotherapy. We compared differences in TMB between patients with high and low RS scores, as well as the correlation between RS score and TMB. The results revealed a negative correlation between RS score and TMB, and patients with low RS scores had higher TMB **(** **Figure 8A****)**. The proportion of patients with MSS and MSI-H in different RS score groups was statistically different. Patients with MSS had higher RS scores than those with MSI **(** **Figure 8B****)**. The TIDE algorithm was used to assess the response to immunotherapy in the high-RS and low-RS score groups, and there was no statistically significant difference between the two groups, despite a huge disparity in median TIDE scores (**Figure 8C**). Furthermore, we discovered significant differences in the T-cell exclusion score, T-cell dysfunction, and MSI between the two risk groups (**Figures 8D–F**) indicating that the low-RS score group may be more susceptible to immunotherapy. Under the AUC, the result illustrated that our risk model was the best compared with TIS and TIDE **(** **Figure 8G****)**. We next validated this hypothesis in the anti-PDL1 immunotherapy cohort (IMvigor210) and discovered that patients in the low-RS score group had a longer survival and a better prognosis than those in the high-RS score group (**Figure 8H**). It was discovered that the low-RS score group had more patients with complete response (CR) and partial response (PR), whereas the high-RS score group had more patients with stable disease (SD) and progressive disease (PD) (**Figure 8I**). In addition, the group of non-responders had a higher RS score **(** **Figure 8J****)**. In conclusion, the RS score can effectively assess the immunotherapy sensitivity of patients.

### Drug sensitivity

**Figure 9:**
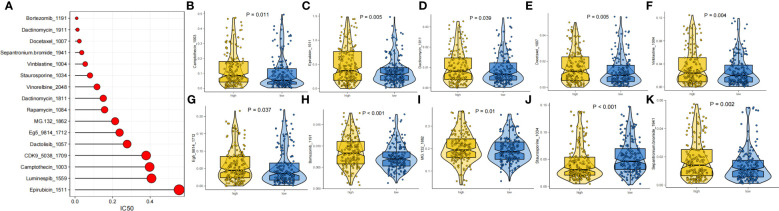
Drug sensitivity analysis. **(A)** IC50 testing results for drugs with IC50<1. **(B-K)** Potential drugs with significant treatment differences in the high- and low-risk subgroups.

The prognosis of GC patients can be improved by selecting an appropriate drug for comprehensive treatment, which still involves chemotherapy and targeted therapies. We investigated the response to different oncology drugs in high-risk and low-risk patients using the “oncoPredict” package. According to the predictive model, the IC50 was predicted for each patient with TCGA-STAD. We identified 16 potential anticancer drugs with IC50<1, indicating a strong inhibitory effect against GC (**Figure 9A**). There were statistically significant differences in the response of 11 drugs in different risk groups (**Figures 9B–K**). Except for staurosporine, most antitumor agents, such as camptothecin, epirubicin, docetaxel, vinblastine, and bortezomib, had lower IC50 values in the low-risk group than in the high-risk group. These findings suggest that patients in the low-risk group were more likely to respond favorably to these medicines.

### Validation of differentially expressed lncRNAs in qRT−PCR

**Figure 10:**
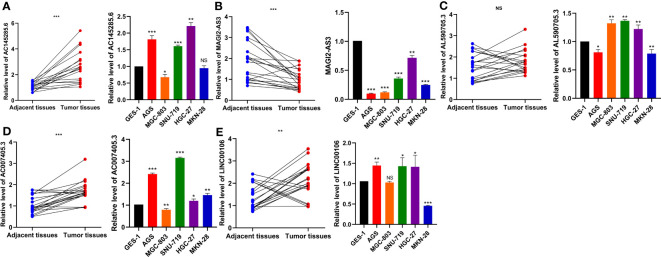
Evaluation of the expression of five DDR-related IncRNAs in GC tissues and cells. **(A)** ACI45285.6 **(B)** MAGIC2-AS3 **(C)** AL590705.3 **(D)** AC007405.3 **(E)** LINCO00106. NS: no significance, *P<0.05, **P<0.01, ***P<0.001.

To verify the expression patterns of the five DDR-related lncRNAs selected in the construction of the risk model, qRT−PCR was used to detect the differential expression of each lncRNA in GC cell lines and tissues. The results showed that the expression of AC145285.6, AC007405.3, and LINC00106 was elevated in GC tissues, whereas the expression of MAGI2-AS3 was decreased. Specifically, AC145285.6 was overexpressed in AGS, SNU-719, and HGC-27 cells but was expressed at low levels in MGC-803 cells (**Figure 10A**). MAGI2-AS3 decreased in all GC cell lines (**Figure 10B**). AL590705.3 was marginally elevated in the MGC-803, SNU-719, and HGC-27 cell lines (**Figure 10C**). AC007405.3 was upregulated in AGS, SNU-719, HGC-27, and MKN-28 cells, while it was downregulated in MGC-803 cells (**Figure 10D**). LINC00106 was elevated in AGS, SNU-719, and HGC-27 cell lines but decreased in MKN-28 cells (**Figure 10E**).

## Discussion

With the advent of personalized medicine, it is crucial to investigate methods for early detection, methods to predict the prognosis, therapeutic sensitivity markers, and potential therapeutic targets to improve the OS rate among GC patients. High DNA damage repair activity in cancer cells causes resistance to chemotherapeutic agents and reduces the efficacy of immunotherapy (37, 38). The development of GC is associated with mutations in oncogenes, cancer suppressor genes, and DDR-related genes, such as KRAS and BRCA1/2, which promote genomic instability and carcinogenesis. Based on DNA damage repair, multiple biomarkers with strong predictive effectiveness for prognosis and anticancer treatment have been identified (39–41). Nevertheless, recent research has mostly focused on the protein-coding genes involved in DDR regulation. It is well known that lncRNAs play a role in the development of GC (42), and there have been multiple studies of lncRNAs to construct GC prognostic models, including ferroptosis (43), pyroptosis (44), necroptosis (45), and immune-related (46) lncRNA prognostic models, many of which have not been validated in external data sets or additional experimental validation. Previous studies have shown that lncRNAs are involved in multi-drug resistance (MDR) in GC, with specific mechanisms including DNA damage repair (47). Therefore, a thorough investigation of the prognostic significance of DDR-related lncRNAs is essential for GC.

Using univariate Cox and LASSO regression in supervised learning, five DDR-related lncRNAs were used to develop a risk score model in this study. In the TCGA-STAD internal cohort, the low-risk group displayed superior OS. In contrast to other clinical characteristics (such as age, grade, and stage), the risk score had a strong predictive influence on prognosis. In addition, by integrating risk score, age, and stage into a nomogram, the results demonstrated that risk score and age could considerably influence GC prognosis, and risk score can be applied as an independent prognostic factor for GC. In addition, we effectively separated the internal cohort of patients into two clusters using a consensus clustering algorithm in unsupervised learning, and we analogously discovered that cluster 2 had higher survival rates than cluster 1. The high-risk group was enriched in signaling pathways related to ECM, tumor invasion, and metastasis, whereas the low-risk group was enriched in pathways related to DDR. Indirectly, the differences in these enrichment pathways imply disparities in prognosis across patients with different risk scores. After establishing the RS score by calculating the differential gene signature A/B of two different risk groups using GSVA, we validated that it could better fit the risk score in the internal cohort and then validated the prognostic value of the RS score using five GSE datasets to broaden the application of the risk model.

The five DDR-related lncRNAs that were used to construct the risk model were differentially expressed in TCGA-STAD, and among them, MAGI2-AS3, LINC00106, and AL590705.3 had prognostic significance. MAGI2-AS3, which can function as a competitive endogenous RNA (ceRNA), was found to be dysregulated in several cancers (48). High MAGI2-AS3 expression was related to poor prognosis in GC, and MAGI2-AS3 overexpression facilitated the migration and invasion of GC cells by sponging miR-141/200a and upregulating ZEB1 expression (49). Notably, MAGI2-AS3 can also affect cancer progression via epigenetic regulation. In breast cancer (BC), for instance, overexpression of MAGI2-AS3 reduces the proliferation and migration of BC cells by downregulating DNA methylation of MAGI2 and blocking the Wnt/-catenin pathway (50). In addition, MAGI2-AS3 expression was downregulated in leukemic stem cells (LSCs), and overexpression of MAGI2-AS3 inhibited LSC self-renewal by inducing TET2 to promote methylation of the LRIG1 promoter region (51). DNA methylation and demethylation are involved in the DNA damage response, and previous research has shown that TET2 plays a crucial role in DDR by modulating the expression of BRCA2 and Lig4 (52). MAGI2-AS3 may bind to CDCA5, RAD51, and UBE2T in GC, as predicted by the DDR-related lncRNA−mRNA network. These genes also play a significant role in DDR (53–55); nonetheless, the mechanism by which MAGI2-AS3 impacts DDR in GC has yet to be elucidated. Previous research revealed that LINC00106 expression was decreased in cisplatin-resistant GC cell lines and that knocking down LINC00106 enhanced the proliferation and migration of AGS cell lines (56). Given that cisplatin induces a DNA damage response by binding to DNA (57), it is possible that LINC00106 is involved in the DDR process and influences the phenotype of GC cells. The remaining three DDR-related lncRNAs have not been researched. We evaluated their expression levels in GC tissues using qRT−PCR, and the results were essentially consistent with those from TCGA-STAD. There was no significant change in AL590705.3 expression, but there were increases in AC145285.6, AC007405.3, and LINC00106 expression and decreases in MAGI2-AS3 expression. Their expression levels in GC cell lines were consistent with those observed in tissues.

Immune evasion, which is a hallmark of cancer cells, hampers and frequently inhibits cancer therapies aimed at stimulating the immune system against malignancy, including defective antigen presentation and immune checkpoint activation, thus leading to immunotherapy resistance (58). A series of DDR-related genes, such as ATM and DNA-PKcs, can facilitate tumor cell immune evasion (5). Alternatively, the DDR pathway is engaged in regulating the functions of immune cells within the tumor microenvironment (TME). It was shown that DNA damage activated the cyclic GMP-AMP synthase-stimulator of interferon genes (cGAS-STING) pathway, resulting in increased tumor-infiltrating lymphocytes (TILs) and IFN-related gene expression (59). Previous research has confirmed that the infiltration of M2 macrophages and mast cells in the TME is associated with a poor prognosis for GC (60, 61). We investigated the infiltration of immune cells in GC in different risk groups and discovered that the levels of M2 macrophages and mast cells climbed in the high-risk group, and their infiltration abundance increased as the risk score increased. These findings indicate that this risk score can distinguish between immune infiltration characteristics of high- and low-risk groups.

Although immune checkpoint inhibitors (ICIs) are a promising strategy for patients with advanced GC, the response rate is still limited, and novel tactics are necessary to maximize the efficacy of ICIs (62). The Cancer Genome Atlas categorized GC into four different molecular subtypes: EBV-positive, MSI-rich, genomically stable, and chromosomally unstable (63). Within these subtypes, EBV-positive and MSI-high tumors have exhibited a higher sensitivity to ICIs. In addition, high TMB has been suggested as a potential biomarker for predicting the clinical efficacy of immunotherapy with ICIs (64). Defective DDR promotes MSI and TMB, whereas a rise in neoantigen burden promotes immunogenicity and attracts more immune cells to the TME. Several studies have found that MUC16 and TTN mutations are associated with better prognosis in GC and higher TMB (65, 66). In TCGA-STAD, MUC16 and TTN mutation rates were higher in the low-risk group. We also observed significant differences between the various RS score groups for several frequently applied immunotherapy biomarkers. MSI and TMB were more significant in the group with a low RS score and negatively correlated with the RS score. Furthermore, the low-RS score group had lower tumor-infiltrating cytotoxic T lymphocyte (CTL) dysfunction and exclusion of CTL levels. Based on these findings, we expected that patients with low RS scores would have a better ICI response, which was confirmed in the immunotherapy cohort. In brief, we discovered that the RS score can distinguish between patients undergoing immunotherapy and may provide a theoretical basis for ICI treatment selection in clinical trials.

It is well established that chemotherapeutics induce DNA damage via direct or indirect pathways, which may contribute to the development of cytotoxicity. If tumor cells can repair such damage, there is a possibility that they will survive chemotherapy or become more tolerant to chemotherapeutic medicines (67). Multiple lncRNAs can impact chemoresistance by regulating DDR-related genes (47). lncRNA-CRAL, for example, inhibits DNA damage and death in cisplatin-resistant GC cells through the miR-505/CYLD/AKT pathway via the ceRNA mechanism and may serve as a biomarker of chemoresistance (68). In this study, we investigated the sensitivity of various drugs in two risk subgroups of patients and found that the low-risk group was more susceptible to both conventional chemotherapeutic treatments and targeted drugs. Low-risk patients respond well pharmacologically to medications that induce DNA damage, inhibit DNA and RNA synthesis, and inhibit spindle formation. In addition, proteasome inhibitors may provide additional benefits for low-risk populations.

Numerous studies have demonstrated the crucial role of the DDR process in regulating the biological behavior and therapeutic susceptibility of tumor cells (69). Risk models based on DDR-related genes have been constructed for different cancers, such as lung adenocarcinoma (LUAD), hepatocellular carcinoma (HCC), and soft tissue sarcoma, with many studies focusing exclusively on the prognostic value of the models (70–72). However, DDR-related lncRNAs have received less attention in GC research. By constructing a DDR-related lncRNA risk model, we not only focused on its predictive significance but also better analyzed the immune infiltration and treatment sensitivity differences among patients. In addition, we found that the risk score/RS score can guide personalized chemotherapy and immunotherapy for patients with GC, thereby improving their prognosis.

Inevitably, the risk model that we constructed has some limitations. First, although the RS score is positively associated with the risk score, the predictive value of the model is indirectly validated by the RS score in the external datasets. In addition, we validated the expression of lncRNAs in the risk model using qPCR, but the specific mechanisms by which DDR-related lncRNAs affect specific DNA repair pathways, the tumor microenvironment, chemotherapy, and immunotherapy sensitivity are unknown and require further validation *in vivo* and *in vitro*.

In conclusion, this risk model is a reliable biomarker for predicting prognosis and treatment sensitivity in GC. Furthermore, this study provides new insights for future basic research and clinical practice in the study of DDR-related lncRNAs.

## Data availability statement

Publicly available datasets were analyzed in this study, the names of the repositories/accession numbers are listed within the article/**Supplementary Material**.

## Ethics statement

The studies involving human participants were reviewed and approved by The Seventh Hospital of Sun Yat-sen University’s Ethical Review Committee. The patients/participants provided their written informed consent to participate in this study.

## Author contributions

ZZ and TM conceived and designed this manuscript. ZZ and YS performed the data analysis and interpreted the data. ZZ and HH performed cell experiments and completed the manuscript. MH and CZ verified the underlying data. All authors contributed to the article and approved the submitted version.
