# Supplementary figures and images for: The DNA damage repair-related lncRNAs signature predicts the prognosis and immunotherapy response in gastric cancer
Source: Front Immunol. 2023 Jun 29;14:1117255. doi: 10.3389/fimmu.2023.1117255 (PMC10339815; doi:10.3389/fimmu.2023.1117255)

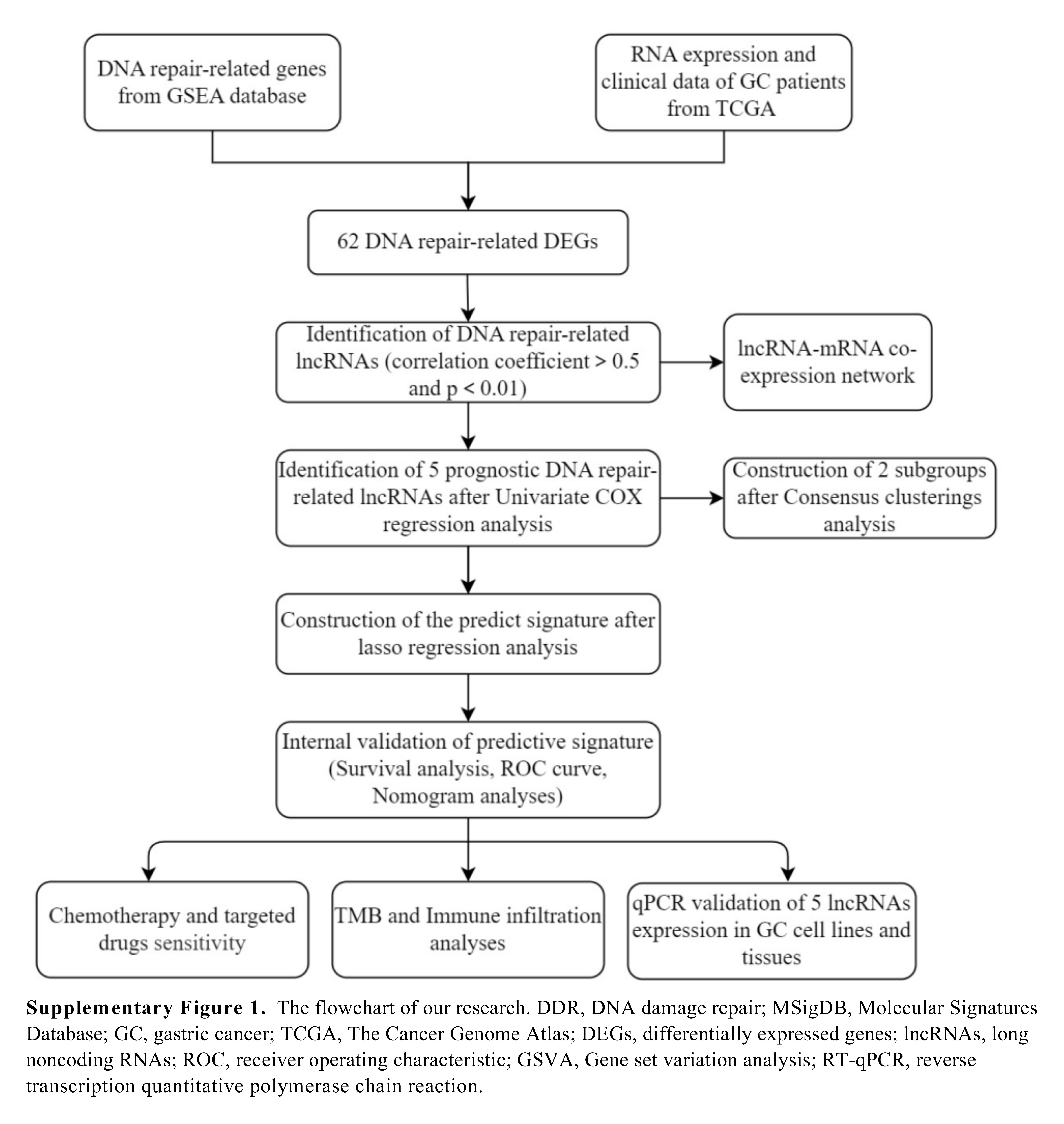

Supplement: Supplementary file 2 [file Image_1.jpeg]

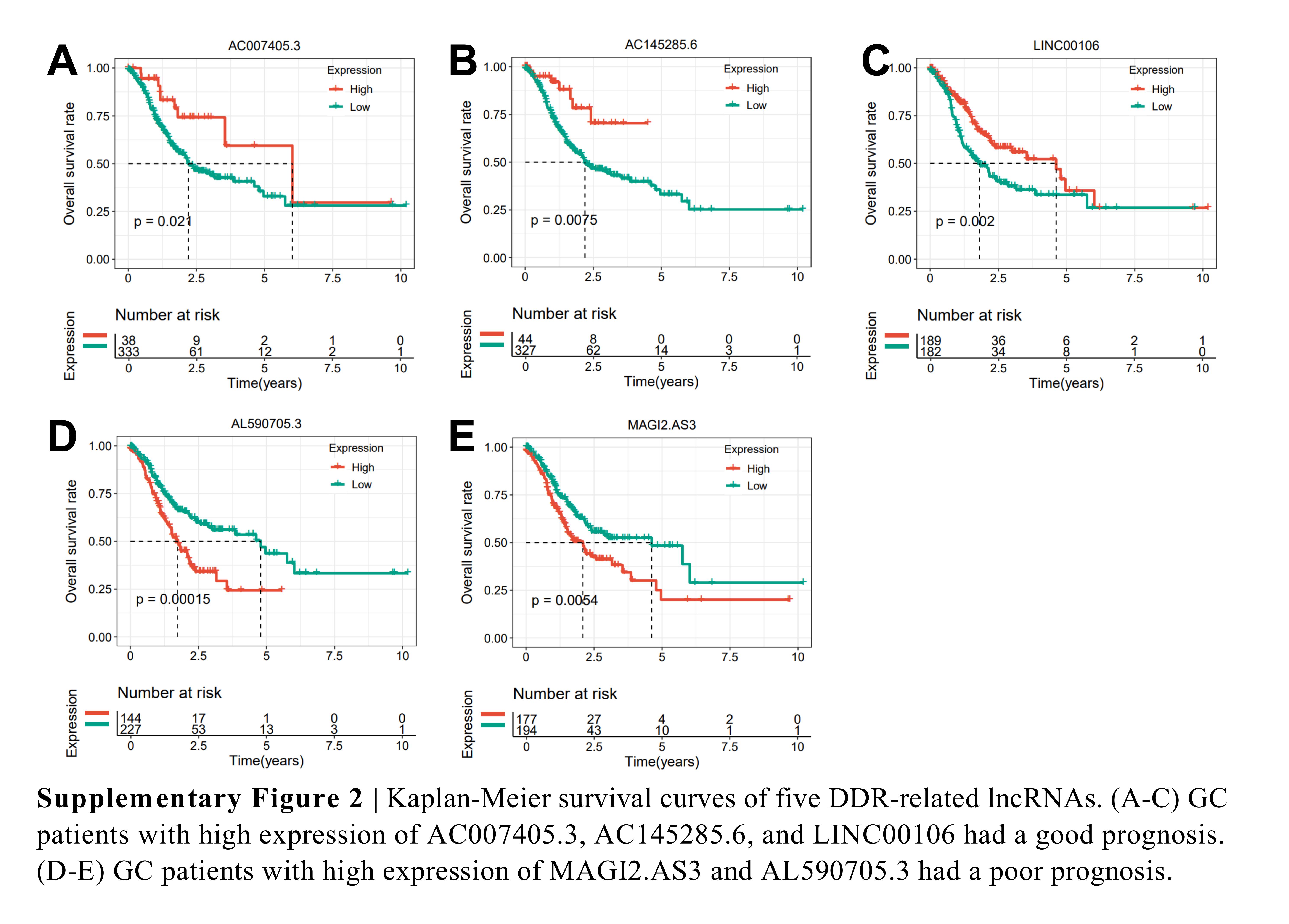

Supplement: Supplementary file 3 [file Image_2.jpeg]

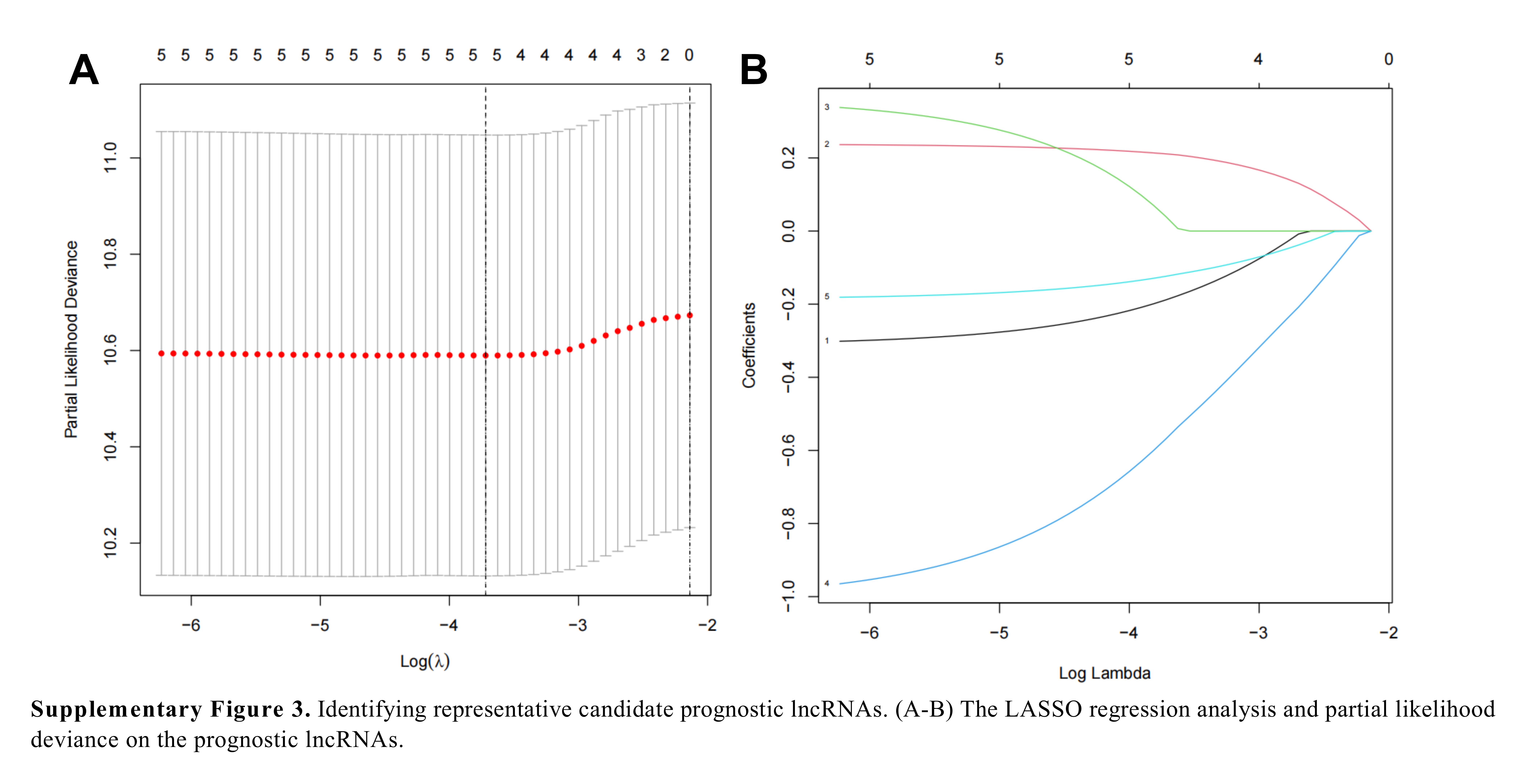

Supplement: Supplementary file 4 [file Image_3.jpeg]

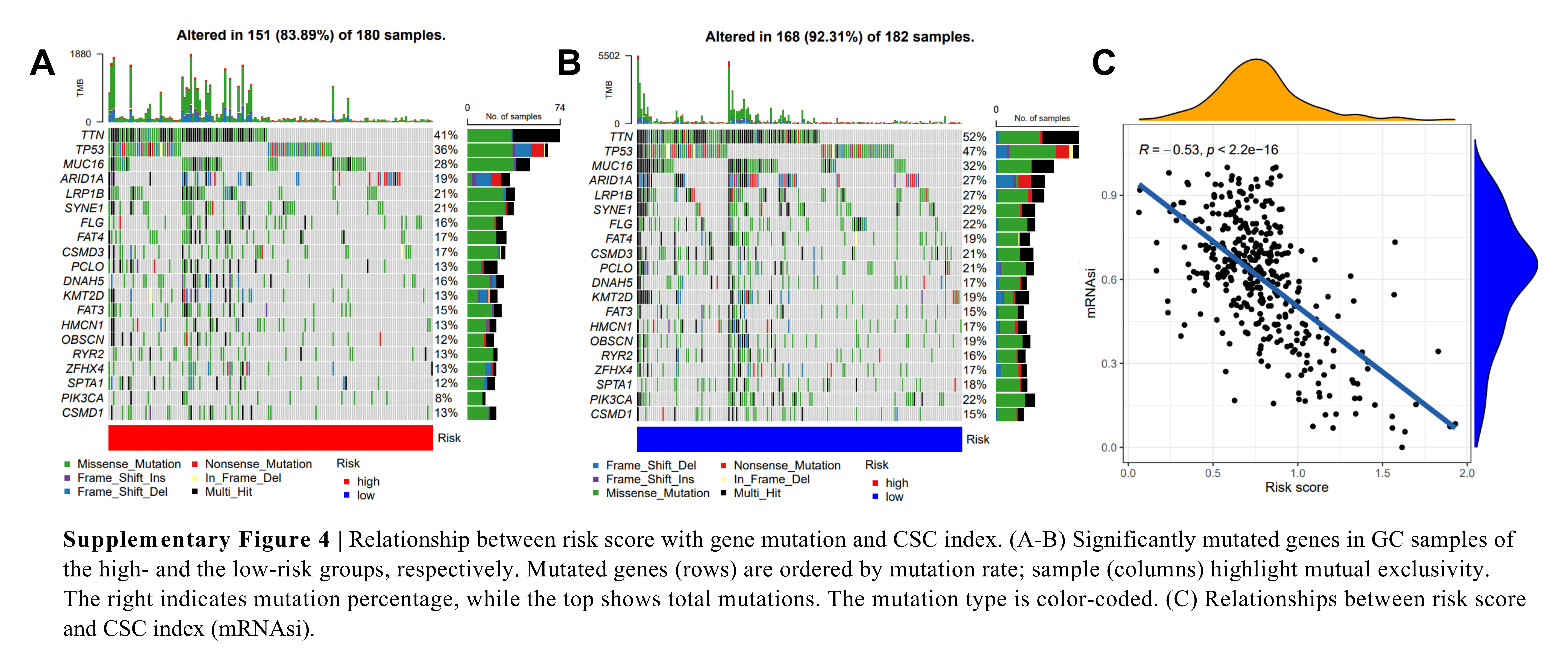

Supplement: Supplementary file 5 [file Image_4.jpeg]
